# Differences in gene expression within a striking phenotypic mosaic *Eucalyptus* tree that varies in susceptibility to herbivory

**DOI:** 10.1186/1471-2229-13-29

**Published:** 2013-02-20

**Authors:** Amanda Padovan, Andras Keszei, William J Foley, Carsten Külheim

**Affiliations:** 1 Research School of Biology, Australian National University, Gould Wing, Building No. 116, ACT 0200, Canberra, Australia

**Keywords:** *Eucalyptus*, Mosaic, Somatic mutation, Comparative transcriptomics, Herbivory

## Abstract

**Background:**

Long-lived trees can accumulate mutations throughout their lifetimes that may influence biotic and abiotic interactions. For example, some *Eucalyptus* trees display marked variation in herbivore defence within a single canopy. These “mosaic” trees support foliage with distinct chemotypes which are differentially favoured by insect and vertebrate herbivores, resulting in susceptible and resistant branches within a single canopy. These mosaic trees provide a unique opportunity to explore the biosynthesis and genetic regulation of chemical defences in the foliage. The biosynthesis of the principal defence compounds, terpenoid-dominated essential oils, is well understood. However, the regulation of the genes involved and thus the control of phenotypic variation within a single tree canopy remains a mystery.

**Results:**

We sequenced the transcriptomes of the leaves of the two different chemotypes of a chemically mosaic *Eucalyptus melliodora* tree using 454 pyrosequencing technology. We used gene set enrichment analysis to identify differentially expressed transcripts and found the proportion of differentially expressed genes in the resistant and susceptible foliage similar to the transcript difference between functionally distinct tissues of the same organism, for example roots and leaves. We also investigated sequence differences in the form of single nucleotide polymorphisms and found 10 nucleotides that were different between the two branches. These are likely true SNPs and several occur in regulatory genes.

**Conclusion:**

We found three lines of evidence that suggest changes to a ‘master switch’ can result in large scale phenotypic changes: 1. We found differential expression of terpene biosynthetic genes between the two chemotypes that could contribute to chemical variation within this plant. 2. We identified many genes that are differentially expressed between the two chemotypes, including some unique genes in each branch. These genes are involved in a variety of processes within the plant and many could contribute to the regulation of secondary metabolism, thus contributing to the chemical variation. 3. We identified 10 SNPs, some of which occur in regulatory genes that could influence secondary metabolism and thus contribute to chemical variation. Whilst this research is inherently limited by sample size, the patterns we describe could be indicative of other plant genetic mosaics.

## Background

Somatic mutations in multicellular organisms can lead to genetically mosaic individuals [1,2]. These mutations occur in a somatic cell line and are due to DNA sequence changes, chromosomal aberrations or epigenetic alterations of DNA [3]. Whereas the genetic changes are interesting, some somatic mutations are truly remarkable because of the striking phenotypic changes that result. Examples include the different coloured flowers of Japanese morning glory (*Pharbitis nil*) [4] or the disease phenotype associated with cancer in humans [3]. Furthermore, many of our horticultural industries are based on somatic mutations: nectarines are a genetic variant of the peach (*Prunus persica*) and have co-occurred on a single tree since 1937 [5].

Theoretical models predict that genetic mosaics should comprise just 5% of a clonal plant population and this rate should be much lower for animals [6]. This is supported by the data on worldwide cancer rates with approximately 0.2% of the human population having a detected cancer [7]. Therefore, genetic mosaics should be hard to identify, particularly if they are not associated with a phenotypic change or if the phenotypic change is cryptic (e.g. a change in chemical composition of the tissue). Very few genetic mosaics have been described among non-clonal plants and those that have are in crop species, for example peach and nectarine [5]. Striking mosaics have been detected in three *Eucalyptus* species; *E. radiata*[8], *E. melliodora*[9] and *E. sideroxylon*[10], which all vary in foliar chemical composition. Long lived forest trees, such as *Eucalyptus,* can accumulate somatic mutations, which may be favourable under certain biotic or abiotic conditions. These mutations may then persist and can influence interactions with other organisms.

Mosaic *Eucalyptus* trees provide a unique opportunity to investigate specific biosynthetic pathways without the usual challenge of variation between individuals. The transcriptome is one of the best places to look for functional genetic differences because it represents expressed genes and varies with changing conditions [11]. The transcriptomes of different tissues of the same individual are qualitatively and quantitatively different [12], as is the transcriptome of the same tissue from different individuals (of the same species) in similar conditions [13]. Despite this, comparative approaches have succeeded in measuring the response of gene expression to specific changes in the environment, such as drought or salinity stress [13,14]. Comparative transcriptomics approaches can employ a variety of technologies to compare and contrast the transcriptomes of two samples, with the aim of identifying pathways or specific genes that differ with the variation in environment [13-15]. This experimental design has become increasingly popular with the advent of next-generation sequencing technologies, and is especially useful for non-model organisms as it does not require a reference genome [16].

**Figure 1:**
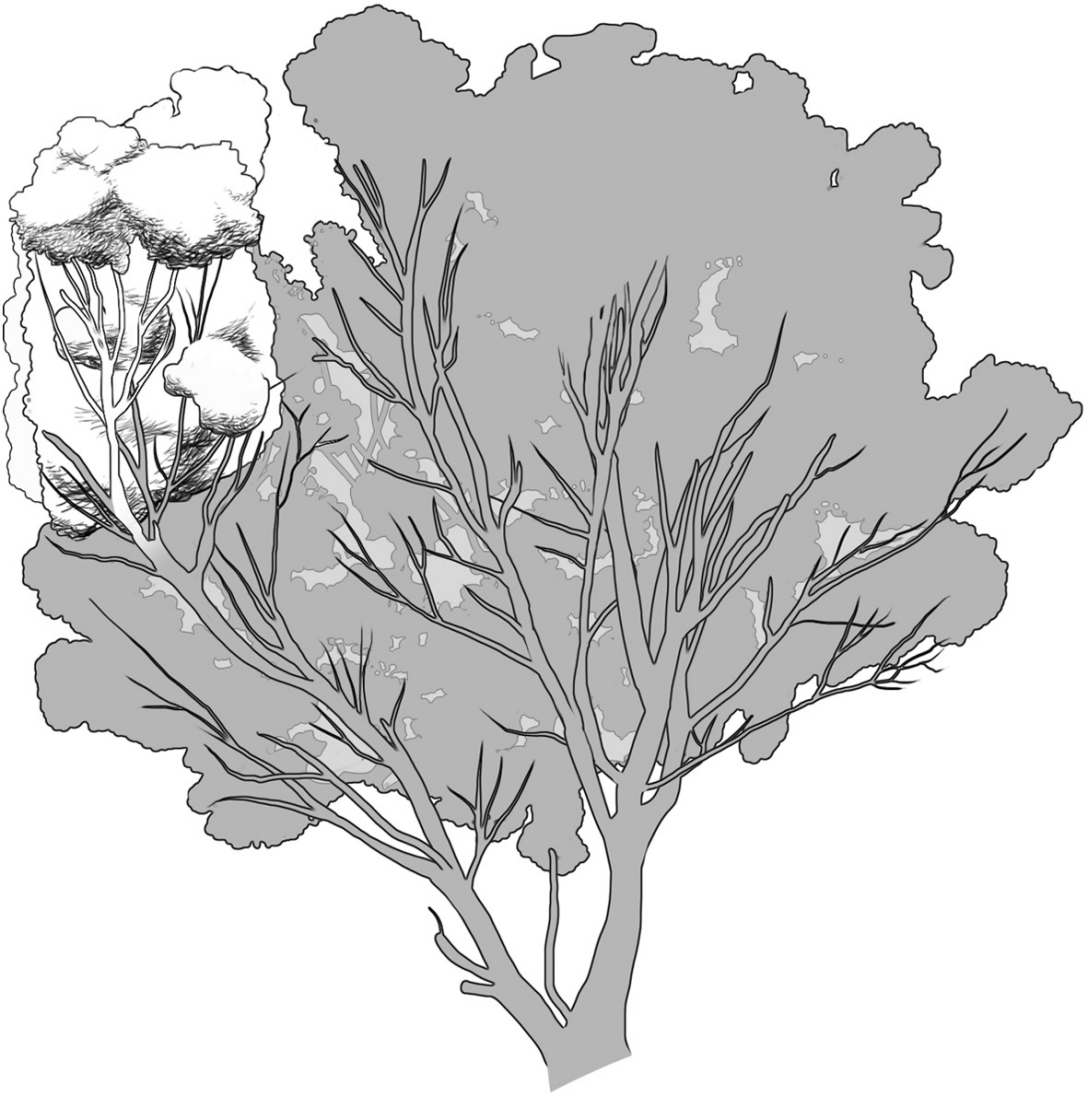
**The mosaic *Eucalyptus melliodora *tree after an insect outbreak (Walsh E, 2012).** The grey area represents foliage susceptible to herbivory, and the white area represents foliage that is resistant to herbivory. Each area has an associated chemical profile [9,17].

We use comparative transcriptomics to investigate differential gene expression, using gene set enrichment analysis (GSEA), between leaves of two chemically different branches of a mosaic *Eucalyptus melliodora* (yellow box) tree. This tree was identified as a phenotypic mosaic in 1990, when Edwards *et al.* reported differential defoliation by insect herbivores: insects defoliated most of the tree (ca 95% defoliation, susceptible chemotype) but left one branch almost untouched (ca 5% defoliation, resistant chemotype – Figure 1) [9]. Padovan *et al.* reported consistent and discontinuous differences in three distinct groups of plant secondary metabolites (monoterpenes, sesquiterpenes and formylated phloroglucinol compounds (FPCs)) between the leaves of the two chemotypes [17], which have persisted since the first chemical profiling was done [9]. The chemical profiles of the resistant and susceptible chemotypes differ significantly in these three biosynthetically distinct classes of secondary metabolites, which supports the prediction of Edwards *et al.* that the chemical patterns observed in the mosaic are due to a somatic mutation in meristematic tissue that was favoured during times of intense herbivory [9].

Here we analyse the transcriptomes of leaves collected from the two chemotypes of the *E. melliodora* mosaic to investigate the functional genetic differences involved in the contrasting susceptibility to herbivory. The specific aims of this study are to:

1. determine if genes of the terpene and FPC biosynthetic pathways are differentially expressed in leaves of susceptible and resistant branches

2. identify the differentially expressed genes and unique genes in the leaves from each branch

3. identify single nucleotide polymorphisms (SNPs) that could be important in determining the susceptible and resistant phenotypes

## Results and Discussion

### Sequencing results and transcriptome assembly

We used 454 technology to sequence the foliar transcriptome of the two chemotypes on the mosaic *E. melliodora* tree, which yielded 277 725 reads (BioSample Project BSH193). Attempts to assemble these reads to the *Eucalyptus grandis* genome sequence with either CLC Genomics Workbench (53% of reads assembled) or Lastz (60% of reads assembled; in Galaxy [18]) were unsatisfactory. Thus we used the *de novo* assembly option in CLC Genomics Workbench to assemble 88% of reads into 13 072 contigs with an average length of 616 bp (Table 1, Additional file 1: Figure S1). Our limited success in assembling the reads against the *E. grandis* genome is probably due to the long reads generated using 454 technology. The software (Lastz and CLC genomics workbench) was optimised to map short reads against a genome and was unable to successfully map the longer reads generated using this method of sequencing.

**Table 1:** **Sequencing statistics generated by *de novo *assembly for the reads generated by sequencing the mRNA pool of leaves from the resistant and susceptible branches of a mosaic *Eucalyptus melliodora***

		**Counts**	**Mean length**	**Total bases**
***de novo *assembly (i)**	total reads	277 725	296	82 179 685
	mapped reads	243 470	290	70 654 843
	unmapped reads	34 255	336	11 524 842
	contigs	13 104	599	7 853 262
**R mapped to *de novo *(ii)**	total reads	133 574	320	42 788 130
	mapped reads	114 093	316	36 061 899
	unmapped reads	19 481	345	6 726 231
	contigs	11 965	615	7 367 368
**S mapped to *de novo *(iii)**	total reads	144 151	273	39 391 555
	mapped reads	130 008	267	34 767 476
	unmapped reads	14 143	327	4 624 079
	contigs	10 130	626	6 350 291

(i) contains all reads from this library, (ii) contains all reads over-represented in the resistant branch compared with the susceptible branch and (iii) contains all reads over-represented in the susceptible branch compared with the resistant branch.

### Identifying the transcripts through BLAST and GO classification

We used Blast2GO [19] to identify the best BLAST hit and to determine the gene ontology (GO) classification for each transcript. There were no BLAST hits for 30% of the contigs (3841 contigs) and no annotation or GO categories could be determined for 9% of the contigs (1199 contigs).

We divided our data into three sets: (i) the reference set which contains all the transcripts sequenced from leaves of the two chemotypes, (ii) all the transcripts over-represented in the leaves of the resistant branch compared with the leaves of the susceptible branch (overR), and (iii) all the transcripts over-represented in the leaves of the susceptible branch compared with the leaves of the resistant branch (overS). Datasets (ii) and (iii) were compiled from fold-change data generated using the expression analysis menu of CLC Genomics Workbench with a minimum threshold of a 2.5 fold difference in expression of the transcripts. For all three datasets most BLAST hits were from *Vitis vinifera* followed by *Populus trichocarpa* and *Arabidopsis thaliana,* (data not shown). Although *Eucalyptus* and *Populus* are more related to each other than to *Vitis*[20] and there are more genes from *Populus* (44 852) than from *Vitis* at NCBI (36 934) – Gene database, National Center for Biotechnology Information, U.S. National Library of Medicine), *Vitis* still provided more BLAST hits than did *Populus*. *Vitis* is rich in secondary metabolites and supports the greatest diversity of terpene synthase genes after *Eucalyptus grandis*[21], which may explain this result. An inherent limitation of this paper is the level of replication; there is no scope for independent replication experiments since the variation occurs within a single tree. Whilst this is a limitation, the work we are doing with this tree contributes significantly to our understanding of the evolution of variation in plant secondary metabolites.

We used the three datasets to address our aims.

#### Are terpene and FPC biosynthetic genes differentially expressed in leaves of the resistant and susceptible chemotypes?

The resistant and susceptible branches of the mosaic tree have a distinct foliar chemical profile with major differences in terpenes and FPCs [17]. Leaves of the resistant chemotype have a higher concentration of monoterpenes and FPCs, while leaves of the susceptible chemotype have a higher concentration of sesquiterpenes (Table 2) [17]. Therefore, we expected to find genes involved in the methyl erythritol pyrophosphate (MEP) pathway and FPC biosynthesis, as well as monoterpene synthases that are significantly up-regulated in the leaves of the resistant chemotype compared with those of the susceptible chemotype. In contrast, genes involved in the mevalonate (MVA) pathway and sesquiterpene synthases should be significantly up-regulated in the leaves of the susceptible chemotype compared with those of the resistant chemotype (for a detailed summary of genes involved in terpene and FPC biosynthesis see Külheim *et al.*[22]). We identified contigs that aligned to genes of interest and compared the relative expression levels in each library. As predicted, in the transcriptome of the resistant leaves there was an over-abundance of transcripts associated with the MEP pathway, as well as an over-abundance of terpene synthases and FPC biosynthetic transcripts, but some individual transcripts were more abundant in the transcriptome of the susceptible leaves.

**Table 2:** **Summary of the within tree foliar chemical variation (adapted from Table**2** by Padovan et al. **[17]**)**

	**Resistant**		**Susceptible**	
	**Concentration**	**No. of Compounds**	**Concentration.**	**No. of Compounds**
Monoterpenes	12.2 (1.18)	10	6.3 (1.55)	15
Sesquiterpenes	1.7 (1.3)	7	24.2 (4.6)	16
FPCs	5.4 (0.3)	3	0.26 (0.3)	3

From each branch (resistant and susceptible) we have reported the concentration and standard deviation across five branches (in mg · g^-1^ dry weight) and number of compounds in the three biosynthetically distinct classes of defence chemicals: monoterpenes, sesquiterpenes and formylated phloroglucinol compounds (FPCs).

We identified 26 different transcript sequences for putative terpene synthase transcripts, using the best BLAST hit, in the entire library of 13,072 contigs: seven monoterpene synthases, 15 sesquiterpene synthases and three triterpene synthases. These transcripts are rare, which makes statistical analyses problematic. However, there are up to 10 putative terpene synthase transcripts that are differentially expressed between the two branches: two triterpene synthases, three monoterpene synthases and five sesquiterpene synthases. Just two terpene synthase transcripts are more abundant in the leaves of the susceptible branch than in the resistant branch: a mono- and a sesquiterpene synthase. This is surprising given that susceptible leaves had a higher concentration of sesquiterpenes and a greater overall diversity of mono- and sesquiterpenes (Table 2).

There are seven genes involved in the MEP pathway, which provides the precursors for monoterpene synthesis [23]. We identified at least two contigs for six of the genes in the MEP pathway. There are no contigs matching *mcs*, the fifth step in the pathway. Four transcripts are more abundant in the transcriptome of the resistant leaves than the susceptible leaves (one copy each of *dxr, mct, cmk, hdr*). Transcripts of these genes correlate strongly with monoterpene production in a closely related tree (*Melaleuca alternifolia*) [24] which is consistent with the higher concentration of monoterpenes in the leaves of the resistant chemotype than leaves of the susceptible chemotype (Table 2).

There are four genes involved in the MVA pathway, which provides the precursors for sesquiterpene synthesis [23] and we identified contigs that align to the final two genes (*mvk* and *pmd*). All of these contigs were equally prevalent in the transcriptomes from the resistant and susceptible leaves. This is surprising since leaves of the susceptible chemotype contain higher concentrations of sesquiterpenes than those of the resistant chemotype, with the opposite pattern found for monoterpenes (Table 2). Several studies have shown that the precursors, IPP and DMAPP, produced by the MEP pathway can be transported across the plastidal membrane and used in sesquiterpene production [24,25]. This could explain the chemical patterns shown for this tree (Table 2).

#### What other genes are differentially expressed between leaves of the resistant and susceptible chemotypes?

The two libraries from the resistant (overR - ii) and susceptible (overS - iii) chemotypes were mapped against the reference sequence of the combined libraries (i). Gene expression in the same tissue taken from the same individual usually shows little or no difference between samples. For example the transcriptome of sperm cells taken from an *Arabidopsis thaliana* plant share 97% identity [26]. Even studies that compare individuals of the same chemotype tend to find few differences [27]: single gene null function mutants in *Arabidopsis* showed little difference in global gene expression [28]. Therefore, we were surprised to find a large proportion of genes differentially expressed between the two chemotypes. We found 436 transcripts (3.3%) that were over-expressed at least 10-fold in the resistant branch, while 970 transcripts (7.4%) were similarly over-expressed in the susceptible branch (Table 2). This difference compares to the variation in gene expression found between functionally distinct tissues in the same organism [29].

**Figure 2:**
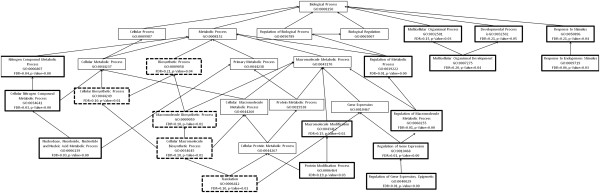
**An enrichment graph of the over- and under-expressed genes in the leaves of the susceptible branch of *Eucalyptus melliodora.*** We have used the gene ontology categories under the biological processes group. These transcripts are 10-fold differentially expressed in leaves of the resistant branch from the same tree. The boxes with a thick border indicate GO categories that are significantly different between the resistant and susceptible branches. Those with a solid outline are significantly up-regulated in the susceptible branch and those with a broken line are significantly down-regulated in the susceptible branch.

**Figure 3:**
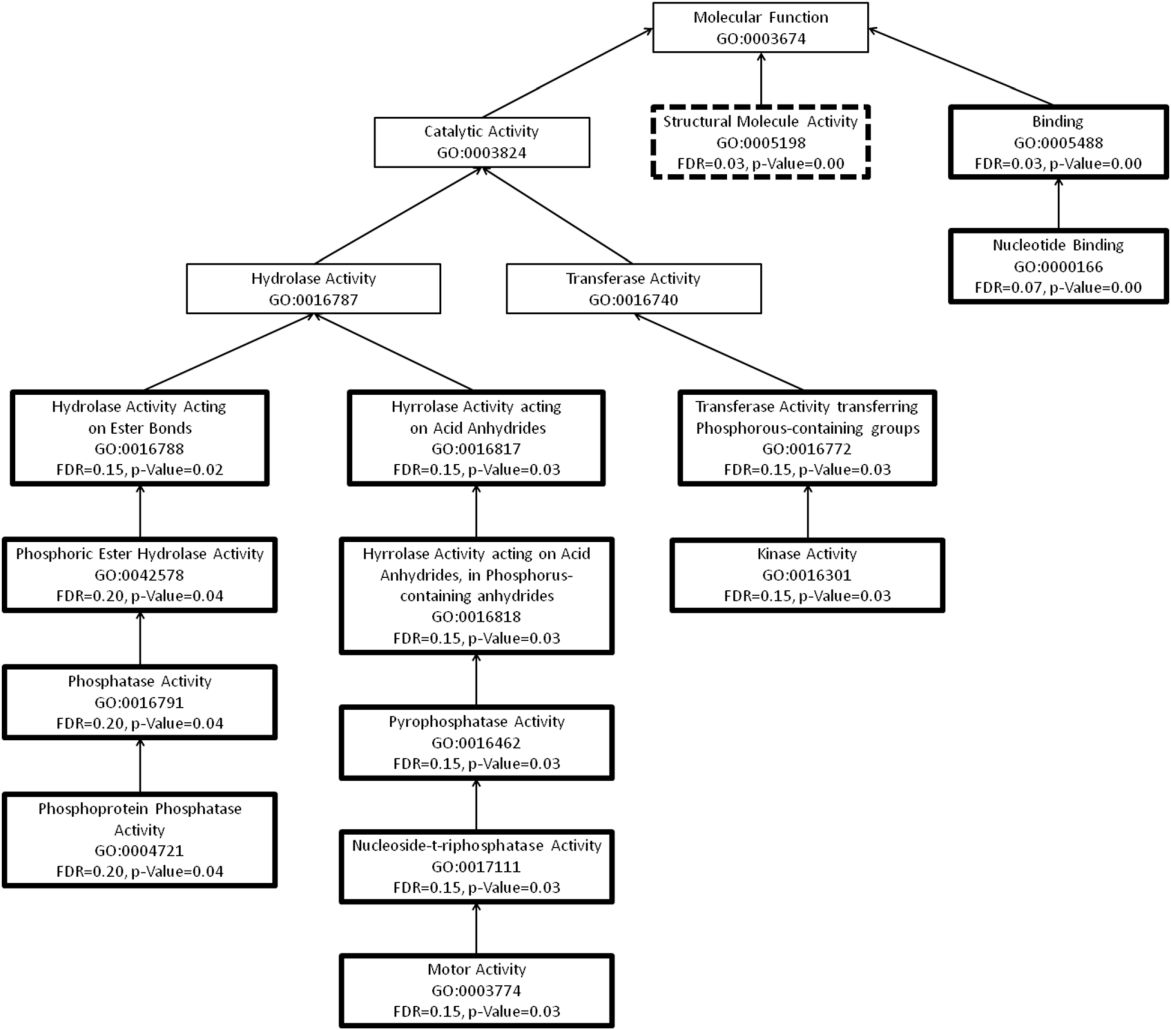
**An enrichment graph of the over- and under-expressed genes in the leaves of the susceptible branch of *Eucalyptus melliodora.*** We have used the gene ontology categories under the molecular function group. These transcripts are 10-fold differentially expressed in leaves of the resistant branch of the same tree. The boxes with a thick border indicate GO categories that are significantly different between the resistant and susceptible branches. Those with a solid outline are significantly up-regulated in the susceptible branch and those with a broken line are significantly down-regulated in the susceptible branch.

**Figure 4:**
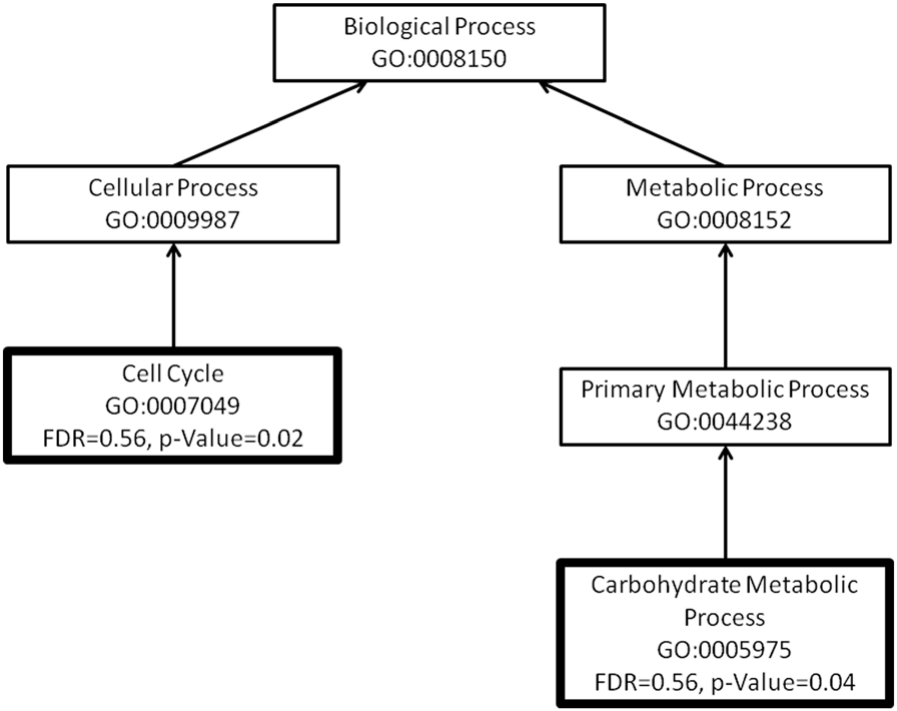
**An enrichment graph of the over- and under-expressed genes in the leaves of the resistant branch of *Eucalyptus melliodora.*** We have used the gene ontology categories under the biological processes group. These transcripts are 10-fold differentially expressed in leaves of the susceptible branch of the same tree. The boxes with a thick border indicate GO categories that are significantly different between the resistant and susceptible branches. Those with a solid outline are significantly up-regulated in the resistant branch and those with a broken line are significantly down-regulated in the resistant branch.

**Figure 5:**
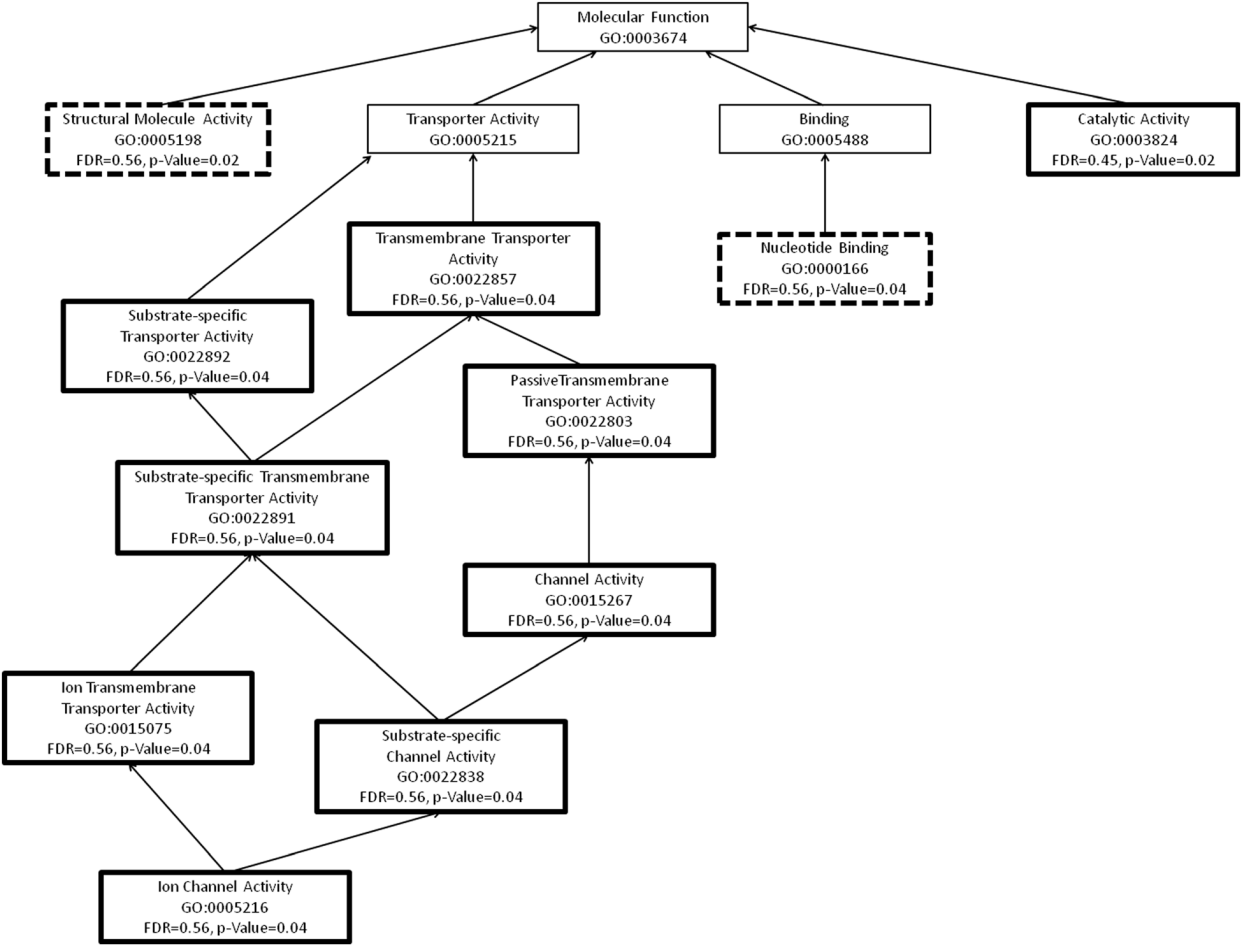
**An enrichment graph of the over- and under-expressed genes in the leaves of the resistant branch of *Eucalyptus melliodora.*** We have used the gene ontology categories under the molecular function group. These transcripts are 10-fold differentially expressed in leaves of the susceptible branch of the same tree. The boxes with a thick border indicate GO categories that are significantly different between the resistant and susceptible branches. Those with a solid outline are significantly up-regulated in the resistant branch and those with a broken line are significantly down-regulated in the resistant branch.

We further investigated specific transcripts that were differentially expressed in the transcriptomes of the two chemotypes. A 2.5 fold threshold and a 10-fold threshold identified the same transcripts as being over and under-represented in the two chemotypes, suggesting that a major reorganisation of gene expression had occurred. The volcano plot (Additional file 2: Figure S2) shows that many genes were significantly differentially expressed between leaves from the resistant and susceptible chemotypes. We used fold-change data, generated in CLC Genomics Workbench, to compile lists of contigs over-expressed in leaves from the resistant and susceptible chemotype. There were many more genes over-expressed in leaves from the susceptible branch (970) compared with leaves from the resistant branch (436). We used Fisher’s Exact Test to determine whether the GO categories of expressed genes were significantly enriched in leaves from the two chemotypes. We used a FDR (false discovery rate)-corrected *P*-value of 0.05 to determine significance of differential expression (Table 3 and Table 4) and the standard *P*-value of significance to show overall patterns of differential gene expression (Figures 2, 3, 4, 5).

**Table 3:** **Statistical analysis (Fisher’s exact test) of the over or under representation of GO categories in the overR (ii) data set derived from the transcriptome of leaves from resistant branches of a mosaic *Eucalyptus melliodora *tree**

**GO Term**	**Name**	**Type**	**FDR**	**p-Value**	**Over/Under**
GO:0003824	catalytic activity	F	0.45	0.00	+
GO:0007049	cell cycle	P	0.56	0.02	+
GO:0005198	structural molecule activity	F	0.56	0.02	-
GO:0005840	ribosome	C	0.56	0.04	-
GO:0030529	ribonucleoprotein complex	C	0.56	0.04	-
GO:0000166	nucleotide binding	F	0.56	0.04	-
GO:0005216	ion channel activity	F	0.56	0.04	+
GO:0015075	ion transmembrane transporter activity	F	0.56	0.04	+
GO:0015267	channel activity	F	0.56	0.04	+
GO:0022803	passive transmembrane transporter activity	F	0.56	0.04	+
GO:0022838	substrate-specific channel activity	F	0.56	0.04	+
GO:0022857	transmembrane transporter activity	F	0.56	0.04	+
GO:0022891	substrate-specific transmembrane transporter activity	F	0.56	0.04	+
GO:0022892	substrate-specific transporter activity	F	0.56	0.04	+
GO:0005975	carbohydrate metabolic process	P	0.56	0.04	+

Type refers to the GO category type: F = molecular function, P = biological processes and C = cellular component. FDR refers to the false discovery rate (FDR)- corrected p-value. p-Value refers to single test p-value. In the over/under column, + signifies over-represented categories and – signifies under-represented categories when compared to the reference data set (i).

**Table 4:** **Statistical analysis (Fisher’s exact test) of the over or under representation of GO categories in the overS (iii) data set derived from the transcriptome of leaves from susceptible branches of a mosaic *Eucalyptus melliodora *tree**

**GO Term**	**Name**	**Type**	**FDR**	**p-Value**	**Over/Under**
GO:0010468	regulation of gene expression	P	0.01	0.00	+
GO:0019222	regulation of metabolic process	P	0.01	0.00	+
GO:0040029	regulation of gene expression, epigenetic	P	0.01	0.00	+
GO:0060255	regulation of macromolecule metabolic process	P	0.01	0.00	+
GO:0005840	ribosome	C	0.01	0.00	-
GO:0030529	ribonucleoprotein complex	C	0.01	0.00	-
GO:0005198	structural molecule activity	F	0.03	0.00	-
GO:0005488	binding	F	0.03	0.00	+
GO:0006139	nucleobase, nucleoside, nucleotide and nucleic acid metabolic process	P	0.04	0.00	+
GO:0006807	nitrogen compound metabolic process	P	0.04	0.00	+
GO:0034641	cellular nitrogen compound metabolic process	P	0.04	0.00	+
GO:0009719	response to endogenous stimulus	P	0.06	0.00	+
GO:0000166	nucleotide binding	F	0.07	0.00	+
GO:0043226	organelle	C	0.07	0.00	-
GO:0005622	intracellular	C	0.09	0.01	-
GO:0043229	intracellular organelle	C	0.09	0.01	-
GO:0044444	cytoplasmic part	C	0.10	0.01	-
GO:0006412	translation	P	0.10	0.01	-
GO:0009059	macromolecule biosynthetic process	P	0.10	0.01	-
GO:0034645	cellular macromolecule biosynthetic process	P	0.10	0.01	-
GO:0044249	cellular biosynthetic process	P	0.10	0.01	-
GO:0044424	intracellular part	C	0.12	0.01	-
GO:0005737	cytoplasm	C	0.13	0.01	-
GO:0044464	cell part	C	0.13	0.01	-
GO:0043412	macromolecule modification	P	0.15	0.02	+
GO:0016788	hydrolase activity, acting on ester bonds	F	0.15	0.02	+
GO:0016301	kinase activity	F	0.15	0.03	+
GO:0016772	transferase activity, transferring phosphorus-containing groups	F	0.15	0.03	+
GO:0003774	motor activity	F	0.15	0.03	+
GO:0016462	pyrophosphatase activity	F	0.15	0.03	+
GO:0016817	hydrolase activity, acting on acid anhydrides	F	0.15	0.03	+
GO:0016818	hydrolase activity, acting on acid anhydrides,	F	0.15	0.03	+
GO:0017111	nucleoside-triphosphatase activity	F	0.15	0.03	+
GO:0032501	multicellular organismal process	P	0.15	0.03	+
GO:0043227	membrane-bounded organelle	C	0.15	0.02	-
GO:0043231	intracellular membrane-bounded organelle	C	0.15	0.02	-
GO:0043228	non-membrane-bounded organelle	C	0.15	0.03	-
GO:0043232	intracellular non-membrane-bounded organelle	C	0.15	0.03	-
GO:0006464	protein modification process	P	0.19	0.03	+
GO:0007275	multicellular organismal development	P	0.20	0.04	+
GO:0004721	phosphoprotein phosphatase activity	F	0.20	0.04	+
GO:0016791	phosphatase activity	F	0.20	0.04	+
GO:0042578	phosphoric ester hydrolase activity	F	0.20	0.04	+
GO:0032991	macromolecular complex	C	0.20	0.04	-
GO:0050896	response to stimulus	P	0.21	0.04	+
GO:0032502	developmental process	P	0.21	0.05	+
GO:0009058	biosynthetic process	P	0.21	0.04	-

Type refers to the GO category type: F = molecular function, P = biological processes and C = cellular component. FDR refers to the false discovery rate (FDR)- corrected p-value and the thick horizontal line shows a significance level of 0.05 FDR. p-Value refers to single test p-value. In the over/under column, + signifies over-represented categories and – signifies under-represented categories when compared to the reference data set (i).

We observed an up-regulation of transcripts with trans-membrane transport and channel activity in the leaves of the resistant chemotype (Figures 2 and 3). Transcripts with nucleotide binding, structural molecular activity and those associated with ribosomes and their complexes were down-regulated in these leaves (Figures 2 and 3). This suggests that metabolites are being transported across membranes [30] and that there is less interaction between nucleic acids and regulators, modifiers and replication machinery resulting in a decrease in overall gene expression (Table 3).

The genes up-regulated in the leaves of the susceptible chemotype, include those involved in the regulation of gene expression and metabolism, macromolecule modification and response to endogenous stimulus (FDR-corrected *P*-value, see Table 4). These include genes with specific transferase, kinase, phosphatase and hydrolase activity (Figures 4 and 5). Genes involved in translation and macromolecular biosynthesis were down-regulated in the leaves of the susceptible chemotype (Figures 4 and 5). This suggests there is a tightly controlled mechanism responding to stimuli that involves changes to gene expression and protein modification in leaves of the susceptible branch. This is expected due to the complexity of the sesquiterpene profile of the susceptible leaves: the oil from these leaves contains 16 sesquiterpenes, compared with seven sesquiterpenes in the foliar oil of the resistant chemotype. Each of these compounds is associated with a gene which must be regulated [31].

We found 455 contigs that are unique to the transcriptome of leaves of the susceptible chemotype and 1645 contigs that are unique to the transcriptome of leaves of the resistant chemotype. The transcriptome of the resistant leaves contain more unique genes, excluding regulatory genes, than does that of the susceptible leaves and since these genes require control, there are also many more regulatory genes in the transcriptome of the resistant chemotype (54:5). The leaves of the resistant chemotype also contain a much higher concentration of FPCs than the leaves of the susceptible chemotype which is represented by the number of unique FPC biosynthesis genes in the transcriptome of the resistant chemotype (8:0). The number of unique terpene biosynthetic genes in the transcriptome of the resistant branch is somewhat surprising given that the leaves of the susceptible chemotype have twice the number of terpenes and the terpene concentration was much higher than the leaves of the resistant chemotype (7:1 - Table 2).

#### Identifying single nucleotide polymorphisms (SNPs) that could play a role in the two chemotypes

**Figure 6:**
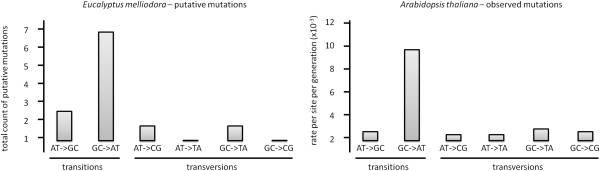
**The spectrum of 10 putative mutations in *E. melliodora *(this study) shows striking similarities with the known spectrum of mutations from a recent mutation accumulation study in *Arabidopsis thaliana *[**[32]**].** On the x-axis we have shown the type of SNP and labelled which polymorphisms are transitions and which are transversions. The y-axis shows the mutation rate in total count of polymorphisms on the left graph (this study) and rate per site per generation on the right graph (*Arabidopsis thaliana* genome).

We found 10 putative single nucleotide polymorphisms (SNPs) between the leaves of the resistant chemotype compared with the leaves of the susceptible chemotype (using CLC Genomics Workbench - Table 5). This is much smaller than the number of SNPs that differ between species: for example, the closely related *E. globulus* and *E. nitens* have 1478 and 1418 SNPs in genes of the secondary metabolism pathway [22] respectively, a difference of 60 SNPs. The proportion of transitions and transversions in these 10 loci (Figure 6) matches the proportions for SNPs in the entire *Arabidopsis* genome [32], making it likely that these are true SNPs. Different mutations occur at different frequencies and the observed frequencies (*E. melliodora*) match the expectations (*Arabidopsis thaliana*). Although the data set is small, it is nevertheless encouraging.

**Table 5:** **The most abundant SNPs with differential representation in the transcriptomes of the resistant (R) and susceptible (S) branches of *Eucalyptus melliodora***

**Contig - position**	**R allele**	**S allele**	**BLAST Hit**
contig 12190 - 233	G/G (28/-)	G/A (25/6)	methyl cpg binding domain 10
contig 5065 - 502	T/C (18/16)	T/T (2/-)	peptidase m1 family protein
contig 5021 - 317	G/G (10/-)	G/A (17/4)	histone deacetylase complex subunit sap18
contig 5063 - 668	T/G (11/8)	T/T (11/-)	pyruvate kinase
contig 7410 - 589	G/A (15/3)	G/G (12/-)	ycf20-like protein
contig 11898 - 31	A/G (15/3)	A/A (12/-)	protein
contig 12260 - 395	C/A (13/2)	C/C (6/-)	-
contig 4834 - 463	C/C (3/-)	C/T (13/2)	-
contig 7446 - 648	G/A (9/7)	G/G (2/-)	protein transport protein sec 13
contig 7510 - 46	G/G (4/-)	G/A (11/3)	-

Contig – position shows the contig number and the nucleotide position that is variable. In both the R allele and S allele columns, the variable nucleotides are written first followed by the number of reads at that position for each variant respectively. BLAST Hit shows the best BLAST Hit for each contig (found using Blast2GO [19]) and – represents contigs that had no best BLAST Hit.

SNPs in two genes, identified by their best BLAST hit, could be involved in epigenetic regulation: methyl cpg binding domain 10 and histone deacetylase complex subunit sap18. Both of these transcripts are more abundant in leaves from the susceptible chemotype than the resistant chemotype and they are homozygous in leaves of the resistant branch, but heterozygous in leaves of the susceptible chemotype. This may be the result of differential allelic expression of certain genes in the resistant leaves compared with the susceptible leaves. In humans, mice and maize, differential allelic expression has been implicated in quantitative trait variation and gene regulation which may result in phenotypic variation [33,34]. These SNPs indicate a role for epigenetic regulation in the formation and maintenance of the mosaic eucalypt and that is an area for future research.

### Are these results representative of other phenotypic mosaics?

Whilst there are only a few examples of phenotypic mosaicism in plants, and our focus has been on *Eucalyptus*, we expect there are many more phenotypic mosaics in natural populations that are yet to be identified. In this report we have focussed on the differences in terpene biosynthetic genes and possible regulatory genes that could explain the chemical variation described [17] and the patterns we have described are likely indicative of gene expression in other plants with similarly high terpene concentrations and variability. However, the idea that the two parts of a mosaic organism have very different gene expression patterns, particularly in those pathways involved in the phenotype, is likely to apply to all phenotypic mosaics.

## Conclusions

Long-lived modular organisms can accumulate somatic mutations over their lifetime enabling them to adjust to a changing environment. Small changes to a ‘master switch’ can lead to large-scale changes to gene expression resulting in the same tissue on the same organism becoming functionally distinct. We found three lines of evidence for this in a mosaic *Eucalyptus melliodora* tree that experiences differential herbivory as a result of chemical diversity: 1. We found differential expression of terpene biosynthetic genes between the two chemotypes that could contribute to chemical variation within this plant. 2. We identified many genes that are differentially expressed between the two chemotypes, including some unique genes in each branch. These genes are involved in a variety of processes within the plant and many could contribute to the regulation of secondary metabolism and thus contribute to the chemical variation between the two chemotypes. 3. We identified 10 SNPs that are likely true SNPs and not artefacts of data collection and analysis. Some of these SNPs occur in regulatory genes that could influence secondary metabolism and thus contribute to foliar terpene variation in this plant. Whilst this research is inherently limited by sample size, the patterns we describe could be indicative of other plant genetic mosaics.

## Methods

### Plant material

Foliage samples were collected at a site at Yeoval (NSW, Australia: 32°45’00.00”S 148°39’00.00”E), known to contain a previously identified as a chemical mosaic *Eucalyptus melliodora*[9,10]. Using a truck-mounted hydraulic-lifted platform, we collected samples of the leaves representing the resistant and susceptible chemotypes of the mosaic. The leaves were immediately put into labelled paper envelopes before being snap frozen in liquid nitrogen. They were subsequently stored at −80°C.

### RNA extraction

The leaves were first ground to a fine powder in liquid nitrogen using a mortar and pestle. We used the QIAgen Plant RNeasy kit to extract RNA (QIAGEN, Valencia California). We followed the manufacturer’s instructions, but added 50 μl of 20% polyvinylpyrrolidone (PVP) and 108 mg of sodium isoascorbate (Na-iASC) to the lysis buffer to remove phenolic compounds and polysaccharides that interfere with RNA extraction and downstream applications [35-37]. We used the Oligotex Direct mRNA Mini Kit (Qiagen, Valencia California) to purify mRNA from these samples, following the manufacturer's instructions.

### cDNA library synthesis

We used the SMARTer RACE cDNA Amplification Kit (Clontech, Mountain View California) to generate a cDNA library following manufacturer’s instructions. We generated one library from leaves of the resistant branch (the resistant library) and one library from leaves of the susceptible branch (the susceptible library). These libraries were not normalised because this may introduce errors in estimating allele frequency and gene expression. Also, low abundance transcripts (like terpene biosynthetic genes) are likely to be lost [38].

### Roche GS FLX Sequencing

cDNA libraries were nebulised and sequenced on a Roche Applied Sciences GS-FLX according to standard procedure (Roche, Indianapolis, IN). The reads were base-called using 454 software, imported into CLC Genomics Workbench (CLC Bio, Denmark) and truncated to remove low quality bases.

### Data Analysis

First, we assembled the library in CLC Genomics Workbench and then assembled the library using Galaxy (Lastz, [18]). In both cases we used the *E. grandis* genome as a reference. With the previous methods leading to limited success (percentage of reads aligned to the reference genome were below 50%), we then used CLC Genomics Workbench to do a *de novo* assembly of the reads and Blast2GO [19] to identify the best BLAST hit and to determine the gene ontology (GO) classification for each transcript, using the default settings. We did an enrichment analysis of the over abundant contigs from both the resistant and susceptible libraries against the total library using the Fishers Exact Test (false discovery rate-corrected *P*-value) and generated enrichment plots (*P*-value) of these data in Blast2Go [19].

### SNP discovery

Using CLC Genomics Workbench (CLC Bio, Denmark), we first identified heterozygous sites within the combined *de-novo* assembly with a minimum of three reads from the minor allele. Then, both assemblies for R and S cDNA libraries were searched at the loci which were identified as heterozygous and identified as heterozygous (same as combined) or homozygous for either allele.

## Competing interests

The authors declare no competing interests.

## Authors’ contributions

AP wrote the manuscript and made significant contributions to data analysis. AK prepared the samples for sequencing and made significant contributions to the manuscript. WJF developed the idea for this study and made significant contributions to the manuscript. CK lead the data analysis and made significant contributions to the manuscript. All authors read and approved the final manuscript.

## Supplementary Material

Additional file 1: Figure S1 A histogram of the length of 13,072 contigs generated from the library of transcripts from *Eucalyptus melliodora* leaves of different chemotypes. The average length is 616 bp. Click here for file

Additional file 2: Figure S2 Volcano plot of transcripts from the leaves of resistant branch of *Eucalyptus melliodora* against the transcripts from the leaves of the susceptible branch of the same tree. The x-axis shows fold change, with positive values representing transcripts over-expressed in the leaves of the resistant branch. The y-axis represents the significance level with a larger value indicating a higher significance. A value of 5 on the y-axis represents a p-value of 0.05. Click here for file
