# Correction: Differences in gene expression within a striking phenotypic mosaic *Eucalyptus* tree that varies in susceptibility to herbivory

**DOI:** 10.1186/1471-2229-13-57

**Published:** 2013-04-02

**Authors:** Amanda Padovan, Robert Lanfear, Andras Keszei, William J Foley, Carsten Külheim

**Affiliations:** 1 Research School of Biology, Australian National University, Gould Wing, Building No. 116, Canberra, ACT, 0200, Australia

## Correction

While compiling the article describing genetic differences between branches of a phenotypic mosaic [1], one of the authors was inadvertently omitted from the author list. This author, Robert Lanfear, has been included in the corrected author list above.

## Authors’ contributions

AP wrote the manuscript and made significant contributions to data analysis. RL developed the novel SNP analysis protocol and made significant contributions to the manuscript. AK prepared the samples for sequencing and made significant contributions to the manuscript. WJF developed the idea for this study and made significant contributions to the manuscript. CK lead the data analysis and made significant contributions to the manuscript. All authors read and approved the final manuscript.
