# Supplementary figures and images for: Differences in gene expression within a striking phenotypic mosaic Eucalyptus tree that varies in susceptibility to herbivory
Source: BMC Plant Biol. 2013 Feb 20;13:29. doi: 10.1186/1471-2229-13-29 (PMC3599227; doi:10.1186/1471-2229-13-29)

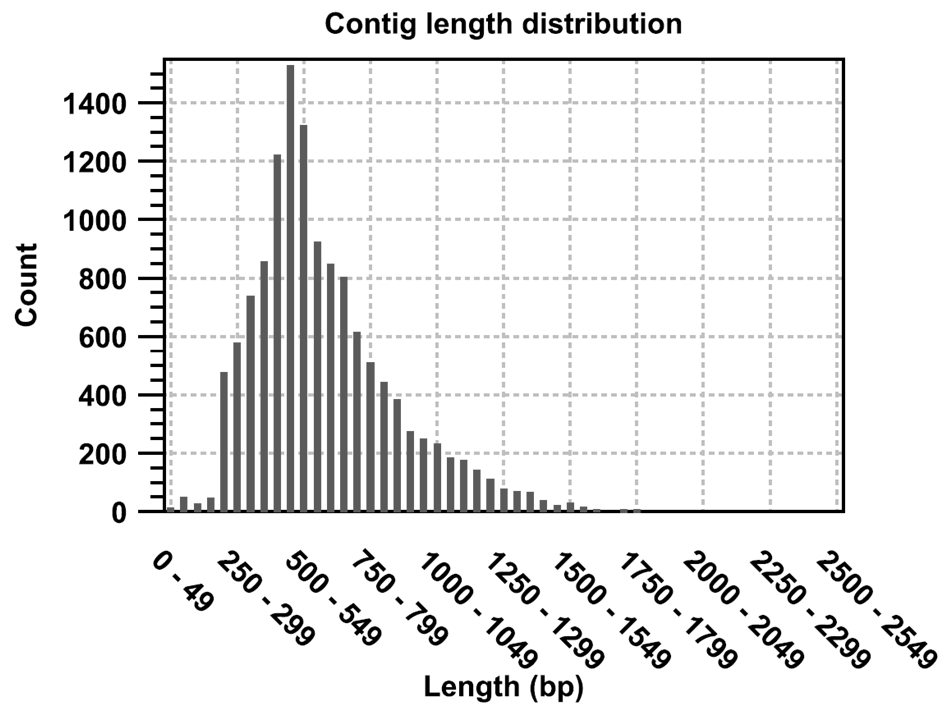

Supplement: Additional file 1: Figure S1 — A histogram of the length of 13,072 contigs generated from the library of transcripts from Eucalyptus melliodora leaves of different chemotypes. The average length is 616 bp. [file 1471-2229-13-29-S1.tiff]

Volcano Plot (Kal's test)

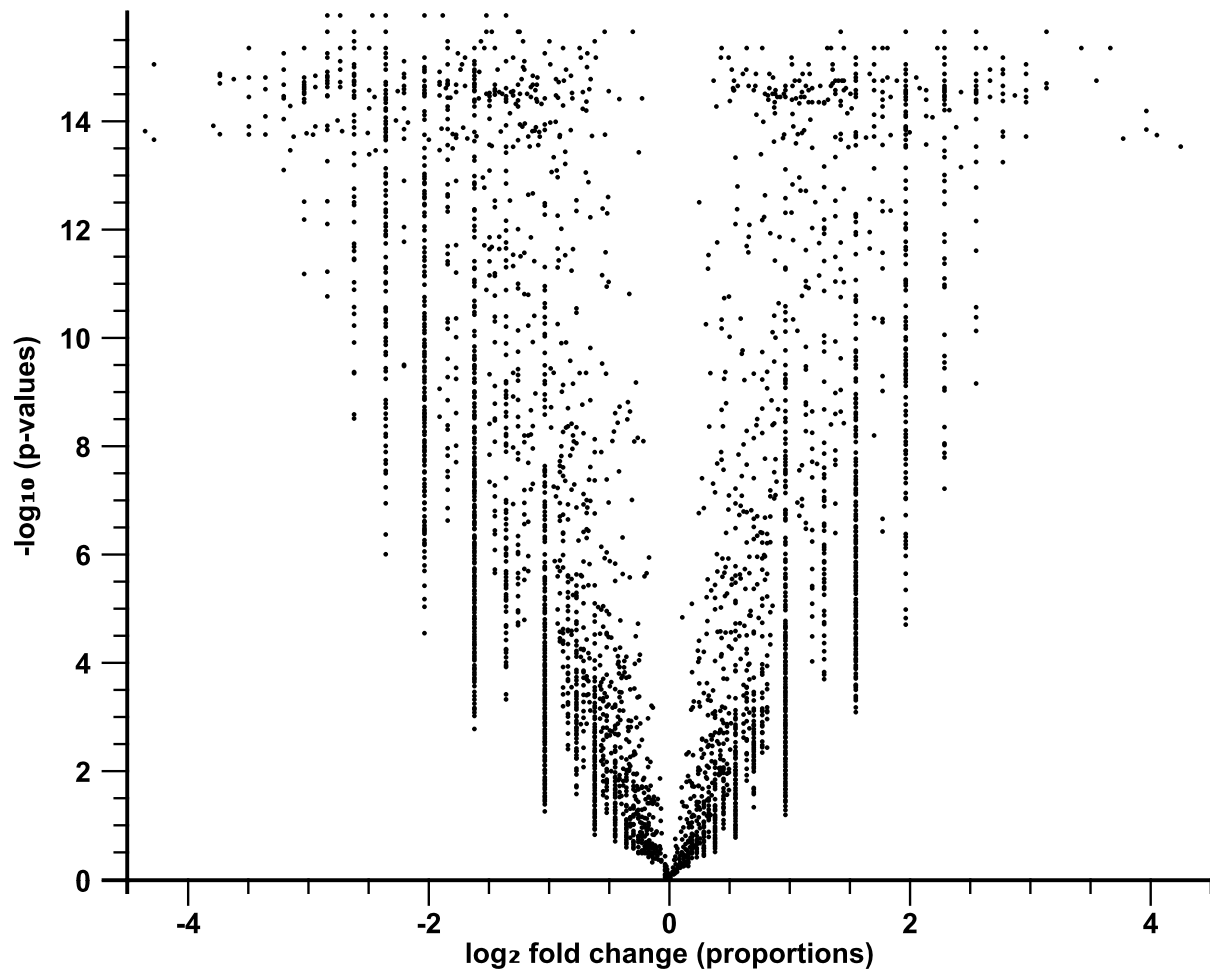

Supplement: Additional file 2: Figure S2 — Volcano plot of transcripts from the leaves of resistant branch of Eucalyptus melliodora against the transcripts from the leaves of the susceptible branch of the same tree. The x-axis shows fold change, with positive values representing transcripts over-expressed in the leaves of the resistant branch. The y-axis represents the significance level with a larger value indicating a higher significance. A value of 5 on the y-axis represents a p-value of 0.05. [file 1471-2229-13-29-S2.pdf]
